# Bayesian causal network modeling suggests adolescent cannabis use accelerates prefrontal cortical thinning

**DOI:** 10.1038/s41398-022-01956-4

**Published:** 2022-05-06

**Authors:** Max M. Owens, Matthew D. Albaugh, Nicholas Allgaier, Dekang Yuan, Gabriel Robert, Renata B. Cupertino, Philip A. Spechler, Anthony Juliano, Sage Hahn, Tobias Banaschewski, Arun L. W. Bokde, Sylvane Desrivières, Herta Flor, Antoine Grigis, Penny Gowland, Andreas Heinz, Rüdiger Brühl, Jean-Luc Martinot, Marie-Laure Paillère Martinot, Eric Artiges, Frauke Nees, Dimitri Papadopoulos Orfanos, Herve Lemaitre, Tomáš Paus, Luise Poustka, Sabina Millenet, Juliane H. Fröhner, Michael N. Smolka, Henrik Walter, Robert Whelan, Scott Mackey, Gunter Schumann, Hugh Garavan, The IMAGEN Consortium, Tobias Banaschewski, Gareth J. Barker, Arun L. W. Bokde, Sylvane Desrivières, Herta Flor, Antoine Grigis, Hugh Garavan, Penny Gowland, Andreas Heinz, Rüdiger Brühl, Jean-Luc Martinot, Marie-Laure Paillère Martinot, Eric Artiges, Frauke Nees, Dimitri Papadopoulos Orfanos, Herve Lemaitre, Tomáš Paus, Luise Poustka, Sarah Hohmann, Sabina Millenet, Juliane H. Fröhner, Michael N. Smolka, Henrik Walter, Robert Whelan, Gunter Schumann

**Affiliations:** 1 Department of Psychiatry, University of Vermont Larner College of Medicine, Burlington, VT USA; 2 Psychiatry Department, University of Rennes 1, Rennes, France; 3 Adult University Psychiatry Department, Guillaume Régnier Hospital, Rennes, France; 4 U1288 Empenn, UMR 6074, IRISA, Rennes, France; 5 Laureate Institute for Brain Research, Tulsa, OK USA; 6 Department of Child and Adolescent Psychiatry and Psychotherapy, Central Institute of Mental Health, Medical Faculty Mannheim, Heidelberg University, Mannheim, Germany; 7 Discipline of Psychiatry, School of Medicine and Trinity College Institute of Neuroscience, Trinity College Dublin, Dublin, Ireland; 8 Centre for Population Neuroscience and Precision Medicine (PONS), Institute of Psychiatry, Psychology & Neuroscience, SGDP Centre, King’s College London, London, UK; 9 Institute of Cognitive and Clinical Neuroscience, Central Institute of Mental Health, Medical Faculty Mannheim, Heidelberg University, Square J5, Mannheim, Germany; 10 Department of Psychology, School of Social Sciences, University of Mannheim, Mannheim, Germany; 11 NeuroSpin, CEA, Université Paris-Saclay, F-91191 Gif-sur-Yvette, France; 12 Sir Peter Mansfield Imaging Centre School of Physics and Astronomy, University of Nottingham, University Park, Nottingham, UK; 13 Department of Psychiatry and Psychotherapy Campus Charité Mitte, Charité-Universitätsmedizin, corporate member of Freie Universität Berlin & Humboldt-Universität zu Berlin, Berlin, Germany; 14 Physikalisch-Technische Bundesanstalt (PTB), Braunschweig and Berlin, Braunschweig, Germany; 15 Institut National de la Santé et de la Recherche Médicale, INSERM U A10 “Trajectoires développementales en psychiatrie”; Université Paris-Saclay, Ecole Normale supérieure Paris-Saclay, CNRS, Centre Borelli, Gif-sur-Yvette, France; 16 Institut National de la Santé et de la Recherce Médicale, INSERM U A10 “Trajectoires développementales & psychiatrie”, University Paris-Saclay, Ecole Normale Supérieure Paris-Saclay, CNRS; Centre Borelli, Gif-sur-Yvette, France; 17 AP-HP. Sorbonne Université, Department of Child and Adolescent Psychiatry, Pitié-Salpêtrière Hospital, Paris, France; 18 Institut National de la Santé et de la Recherche Médicale, INSERM U1299 “Trajectoires développementales en psychiatrie”; Ecole Normale supérieure Paris-Saclay, Université Paris-Saclay, Université de Paris, Centre Borelli; Gif-sur-Yvette, & Department of Psychiatry, EPS Barthélémy Durand, Etampes, France; 19 Institute of Medical Psychology and Medical Sociology, University Medical Center Schleswig Holstein, Kiel University, Kiel, Germany; 20 Institut des Maladies Neurodégénératives, CNRS UMR 5293, Université de Bordeaux, Centre Broca Nouvelle-Aquitaine, Bordeaux, France; 21 Departments of Psychiatry and Neuroscience, Faculty of Medicine and Centre Hospitalier Universitaire Sainte-Justine, University of Montreal, Montreal, QC Canada; 22 Departments of Psychology, University of Toronto, Toronto, ON Canada; 23 Department of Psychiatry, University of Toronto, Toronto, ON Canada; 24 Department of Child and Adolescent Psychiatry and Psychotherapy, University Medical Centre Göttingen, von-Siebold-Str. 5, 37075 Göttingen, Germany; 25 Department of Psychiatry and Neuroimaging Center, Technische Universität Dresden, Dresden, Germany; 26 School of Psychology and Global Brain Health Institute, Trinity College Dublin, Dublin, Ireland; 27 Centre for Population Neuroscience and Stratified Medicine (PONS), Department of Psychiatry and Psychotherapy, Charité Universitätsmedizin Berlin, Berlin, Germany; 28 Centre for Population Neuroscience and Precision Medicine (PONS), Institute for Science and Technology of Brain-inspired Intelligence (ISTBI), Fudan University, Shanghai, China; 29 Department of Child and Adolescent Psychiatry and Psychotherapy, Central Institute of Mental Health, Medical Faculty Mannheim, Heidelberg University, Square J5, 68159 Mannheim, Germany; 30 Department of Neuroimaging, Institute of Psychiatry, Psychology & Neuroscience, King’s College London, London, UK; 31 Discipline of Psychiatry, School of Medicine and Trinity College Institute of Neuroscience, Trinity College Dublin, Dublin, Ireland; 32 Centre for Population Neuroscience and Precision Medicine (PONS), Institute of Psychiatry, Psychology & Neuroscience, SGDP Centre, King’s College London, London, UK; 33 Institute of Cognitive and Clinical Neuroscience, Central Institute of Mental Health, Medical Faculty Mannheim, Heidelberg University, Square J5, Mannheim, Germany; 34 Department of Psychology, School of Social Sciences, University of Mannheim, 68131 Mannheim, Germany; 35 NeuroSpin, CEA, Université Paris-Saclay, F-91191 Gif-sur-Yvette, France; 36 Departments of Psychiatry and Psychology, University of Vermont, 05405 Burlington, VT USA; 37 Sir Peter Mansfield Imaging Centre School of Physics and Astronomy, University of Nottingham, University Park, Nottingham, UK; 38 Department of Psychiatry and Psychotherapy CCM, Charité – Universitätsmedizin Berlin, corporate member of Freie Universität Berlin, Humboldt-Universität zu Berlin, and Berlin Institute of Health, Berlin, Germany; 39 Physikalisch-Technische Bundesanstalt (PTB), Braunschweig and Berlin, Braunschweig, Germany; 40 Institut National de la Santé et de la Recherche Médicale, INSERM U A10 “Trajectoires développementales en psychiatrie”; Université Paris-Saclay, Ecole Normale supérieure Paris-Saclay, CNRS, Centre Borelli, Gif-sur-Yvette, France; 41 Institut National de la Santé et de la Recherce Médicale, INSERM U A10 “Trajectoires développementales & psychiatrie”, University Paris-Saclay, Ecole Normale Supérieure Paris-Saclay, CNRS; Centre Borelli, Gif-sur-Yvette, France; 42 AP-HP. Sorbonne Université, Department of Child and Adolescent Psychiatry, Pitié-Salpêtrière Hospital, Paris, France; 43 Institut National de la Santé et de la Recherche Médicale, INSERM U A10 “Trajectoires développementales en Psychiatrie”; Université Paris-Saclay, Ecole Normale supérieure Paris-Saclay, CNRS, Centre Borelli, Gif-sur-Yvette; and Psychiatry Department, EPS Barthélémy Durand, Etampes, France; 44 Institute of Medical Psychology and Medical Sociology, University Medical Center Schleswig Holstein, Kiel University, Kiel, Germany; 45 Institut des Maladies Neurodégénératives, UMR 5293, CNRS, CEA, Université de Bordeaux, 33076 Bordeaux, France; 46 Department of Psychiatry, Faculty of Medicine and Centre Hospitalier Universitaire Sainte-Justine, University of Montreal, Montreal, QC Canada; 47 Departments of Psychiatry and Psychology, University of Toronto, Toronto, ON Canada; 48 Department of Child and Adolescent Psychiatry and Psychotherapy, University Medical Centre Göttingen, von-Siebold-Str. 5, 37075 Göttingen, Germany; 49 Department of Psychiatry and Neuroimaging Center, Technische Universität Dresden, Dresden, Germany; 50 School of Psychology and Global Brain Health Institute, Trinity College Dublin, Dublin, Ireland; 51 Centre for Population Neuroscience and Stratified Medicine (PONS), Department of Psychiatry and Psychotherapy, Charité Universitätsmedizin Berlin, Berlin, Germany; 52 Centre for Population Neuroscience and Precision Medicine (PONS), Institute for Science and Technology of Brain-inspired Intelligence (ISTBI), Fudan University, Shanghai, China

**Keywords:** Molecular neuroscience, Addiction

## Abstract

While there is substantial evidence that cannabis use is associated with differences in human brain development, most of this evidence is correlational in nature. Bayesian causal network (BCN) modeling attempts to identify probable causal relationships in correlational data using conditional probabilities to estimate directional associations between a set of interrelated variables. In this study, we employed BCN modeling in 637 adolescents from the IMAGEN study who were cannabis naïve at age 14 to provide evidence that the accelerated prefrontal cortical thinning found previously in adolescent cannabis users by Albaugh et al. [1] is a result of cannabis use causally affecting neurodevelopment. BCNs incorporated data on cannabis use, prefrontal cortical thickness, and other factors related to both brain development and cannabis use, including demographics, psychopathology, childhood adversity, and other substance use. All BCN algorithms strongly suggested a directional relationship from adolescent cannabis use to accelerated cortical thinning. While BCN modeling alone does not prove a causal relationship, these results are consistent with a body of animal and human research suggesting that adolescent cannabis use adversely affects brain development.

## Introduction

There is substantial evidence that adolescent cannabis use is associated with poorer cognitive function [2], as well as differences in brain structure and function [3]. However, evidence is mixed as to whether the cognitive and neurobiological differences identified are caused by adolescent cannabis use or are pre-existing and might dispose individuals to be more likely to initiate cannabis use. Research in humans suggests that long-term differences in prefrontal morphometry in adolescents using cannabis are most pronounced in regions of the brain that show the highest levels of expression for the cannabinoid receptor 1 gene (CNR1) [4]. Additionally, animal research has shown that THC exposure in adolescent rats alters the morphological trajectory of pyramidal neurons in the prefrontal cortex [5]. However, adult and older adolescent twin studies have found comparable cognitive function in monozygotic twins discordant for cannabis use, though these studies have not investigated brain structure or function [6, 7].

A recent paper by Albaugh et al. [1] found that, in a sample of 799 adolescents, initiation of cannabis use between the ages of 14 and 19 was associated with a higher rate of cortical thinning during that period. In that study, there were no differences in cortical thickness among the groups at age 14 and the subsequent cortical thinning was associated with amount of cannabis used from 14 to 19 in a dose-dependent pattern. It also found that regions demonstrating cannabis-related cortical thinning had, on average, greater availability of CB1 receptors (as assessed by Positron Emission Tomography in a separate sample of young adults), suggesting that the accelerated thinning may be mediated, in part, by cannabis exposure affecting the brain’s endogenous cannabinoid system. That said, it remains possible that these brain changes may not be a consequence of the cannabis exposure but may reflect instead a neurodevelopmental trajectory caused by other factors that is related to a higher likelihood of adolescent cannabis use.

Cortical thinning in adolescence is well-established as a normal trajectory of brain development. Studies estimate cortical thinning of around 1% annually, which comes out to around 0.03–0.06 millimeters per year [8–11]. There is also work showing that negative early life experiences are associated with premature thinning in adolescence [12], which is, in turn, associated with negative outcomes, including greater likelihood of attention-deficit/hyperactivity symptoms [13] and symptoms of depression [14]. Likewise, Albaugh et al. found an association between accelerated cortical thinning and attentional impulsivity, particularly in the DPFC region identified as being related to cannabis use [1]. In total, this literature suggests that accelerated prefrontal thinning resulting from cannabis use is likely to be a negative prognostic factor for mental health outcomes.

Bayesian causal network (BCN) modeling is a method for estimating the directional relationships between a set of differentially related variables [15]. This approach models the relationships among variables as a directed acyclic graph using the conditional dependencies among variables. The structure of a BCN can be estimated through use of structure learning algorithms. These algorithms apply an emerging understanding of how directional relationships are predicated on conditional dependence to determine the BCN that best represents the joint probability distributions of a dataset [16]. Assuming that there are no hidden variables unaccounted for, these networks can be interpreted as implying causal relationships [17]. This approach has recently been used to test the direction of effect between binge drinking and prefrontal and temporal gray matter development, finding that atypical gray matter development appeared to be increasing the likelihood of binge drinking rather than binge drinking producing cortical atrophy [18].

The current study used the same BCN modeling approach as Robert et al. [18] with the same IMAGEN data used in Albaugh et al. [1], to assess whether initiation of cannabis use and changing cortical thickness across adolescence are causally related. We did this by including all variables in the analyses of Albaugh et al., as well as several other potential confounders as variables in a BCN. Specially, in our BCN models we included genetic factors putting individuals at risk for cannabis use, demographic factors, life history, psychopathology, and other substance use. Inclusion of this large set of variables was ideal for the analytic strategy planned, as BCN learning algorithms operate best on many interrelated variables, which allows them to determine the conditional dependencies among them.

## Methods

### Participants and procedures

Participants were drawn from the IMAGEN study conducted across eight European sites. Local ethics research committees approved the study at each site (London, England: Psychiatry, Nursing and Midwifery Research Ethics Subcommittee, Waterloo Campus, King’s College London; Nottingham, England: University of Nottingham Medical School Ethics Committee; Mannheim, Germany: Medizinische Fakultaet Mannheim, Ruprecht Karl Universitaet Heidelberg and Ethik-Kommission II an der Fakultaet fuer Kliniksche Medizin Mannheim; Dresden, Germany: Ethikkommission der Medizinischen Fakultaet Carl Gustav Carus, TU Dresden Medizinische Fakultaet; Hamburg, Germany: Ethics Board, Hamburg Chamber of Physicians; Paris, France: CPP IDF VII (Comité de protection des personnes Ile de France), ID RCB: 2007-A00778-45 September 24, 2007; Dublin, Ireland: TCD School of Psychology REC; and Berlin, Germany: Ethics Committee of the Faculty of Psychology). Written consent was obtained from the adolescent’s parent or guardian, and verbal assent was obtained from the adolescent.

The IMAGEN study included 2223 adolescents recruited at age 14 for MRI, genotyping, and self-report data collection who were assessed again 5 years later using the same battery. The final sample of Albaugh et al. (*N* = 799) was used in the current study, with 162 participants removed for lacking one or more of the additional measures added to this analysis. This resulted in a final sample of 637 participants, which did not differ on any demographic or drug use measure from the sample of Albaugh et al. (*p* > 0.05). Of note, we also repeated analyses in the exact sample from Albaugh et al. (*N* = 799) using only the variables included in that study, finding very similar results (shown in Supplementary Fig. 1 and Supplementary Table 1). Of note, participants were selected for the Albaugh et al. [1] study such that all were cannabis naïve at age 14.

### Measures

#### European school survey project on alcohol and drugs

Cannabis use and tobacco use were assessed at age 14 and age 19 with the ESPAD [19], a self-report questionnaire regarding the use of alcohol, nicotine, and cannabis as well as other substances. Participants indicated how many times they had used each of the substances in their lifetime, in the past 12 months, in the past 30 days, and in the past 7 days using a 7-point scale (where 0 indicates never; 1, 1–2 times; 2, 3–5 times; 3, 6–9 times; 4, 10–19 times; 5, 20–39 times; and 6, ≥40 times). However, since all participants were selected to be cannabis naïve at age 14, only cannabis use at age 19 was included in BCN modeling and other analyses.

#### Alcohol Use Disorders Identification Test

The Alcohol Use Disorders Identification Test (AUDIT) is a 10-item alcohol screening tool that assesses alcohol consumption, drinking behaviors, and alcohol-associated problems [20]. AUDIT was administered to youths at age 14 and 19. The AUDIT Alcohol Consumption scale (AUDIT-C) was used in the present study and is composed of items on AUDIT that explicitly assess the amount and frequency of alcohol consumption.

#### Childhood Trauma Questionnaire

The Childhood Trauma Questionnaire (CTQ) is an assessment of adverse experiences occurring during childhood with good validity and internal consistency [21]. This self-report measure contains 70 items that make up five factors: physical abuse, emotional abuse, sexual abuse, physical neglect, and emotional neglect. Responses are on a 5-point Likert-type scale according to the frequency with which an experience occurred, with 1 = “never true” and 5 = “very often true.” In the current study, we summed the five factors to create a total score.

#### Strengths and Difficulties Questionnaire

The Strengths and Difficulties Questionnaire (SDQ) was used to assess symptoms of hyperactivity and inattention [22], which we used as a measure of Attention Deficit/Hyperactivity Disorder (ADHD) symptoms. The SDQ is a reliable and valid measure of youth emotional and behavior symptoms; scores on the SDQ are predictive of increased probability of clinician-rated psychiatric disorders and it has good retest stability over 4–6 months [23].

#### Substance Use Risk Profile Scale

The Substance Use Risk Profile Scale (SURPS) was used to assess personality traits related to substance use [24]. The SURPS is a 23-item 4-point Likert scale measure that assesses four personality variables: anxiety sensitivity, introversion/hopelessness, impulsivity, and sensation-seeking. From this measure, the trait sensation seeking was selected for BCN modeling, as it was the trait with the largest bivariate relationship with cannabis use at age 19 (*r* = 0.29 at age 19).

#### Pubertal Development Scale

Pubertal status was assessed using the pubertal development scale, a self-report measure completed by the participant. This measure has been shown to have good reliability and to correspond with accepted self-report and biological measures of pubertal development [25].

#### Socioeconomic status

A composite of socioeconomic status (SES) was derived by aggregating: Mother’s Education Score, Father’s Education Score, Family Stress Unemployment Score, Financial Difficulties Score, Home Inadequacy Score, Neighborhood Score, Financial Crisis Score, Mother Employed Score, and Father Employed Score in a manner consistent with prior work in the IMAGEN dataset [26].

#### Demographic questionnaire

Children’s age, sex, and handedness were measured using a demographic questionnaire completed by their parent/guardian.

#### Structural MRI

Structural magnetic resonance image (MRI) data were acquired with a 3-dimensional T1-weighted magnetization prepared gradient echo sequence based on the one used in the Alzheimer’s Disease Neuroimaging Initiative protocol [27]. These T1 images were processed using the CIVET pipeline (v2.1.0) on the CBRAIN platform using Compute Canada. In the current project, average cortical thickness was extracted from the regions of interest identified in Albaugh et al. that showed a dose-dependent response to cannabis use. This region spanned the left and right dorsomedial and dorsolateral prefrontal cortices and is hereafter referred to as the dorsal prefrontal cortex (DPFC).

For consistency with prior work using BCN modeling in the IMAGEN dataset [18], we used a principal components analysis to create a single variable for cortical thickness in both hemispheres. All variables (cortical thickness, cannabis use, other substance use, age, puberty, polygenic risk score for cannabis use, childhood trauma, socioeconomic status, handedness, and ADHD symptoms) were residualized for site and sex. This single variable was derived from the first principal component. This was done for consistency with recent work using BCN modeling focused on alcohol and brain morphometry in the IMAGEN dataset [18]. This was done for DPFC thickness at age 14 (eigenvalue = 1.8; percent of variance explained = 92%) and the change in DPFC from ages 14 to 19 (eigenvalue = 1.7; percent of variance explained = 87%).

#### Polygenic risk score

In calculating a polygenic risk score (PRS) for cannabis, genotypes were imputed using the 1000 Genomes Project phase 3 reference panel for Europeans using the Michigan Imputation Server [28]. SNPs that did not meet quality control criteria (Minor Allele Frequency < 0.01; Genotype Call Rate < 95%; Hardy–Weinberg Equilibrium < 1 × 10^−6^) were excluded. Genetic variants imputed with lower accuracy (*R*^2^ < 0.6), insertion/deletions and palindromic SNPs were excluded, resulting in 5,183,147 SNPs. The PRS was calculated based on a genome-wide association study (GWAS) conducted in an independent sample. This GWAS described the genetic correlates of total lifetime frequency of cannabis use using data from the International Cannabis Consortium, UK Biobank, and 23andMe datasets [29]. All participants (53,179 cases and 131,586 controls) included in the GWAS were of European Ancestry, as are all participants in the present analysis. This PRS was calculated using PLINK 2.0 [30]. Index variants were identified by clumping using an r2 threshold of 0.1 with a 1000 kb window using the 1000 Genomes (EUR) as reference. The available summary statistics for this GWAS included 11,535,788 SNPs. After quality control procedures (i.e., removal of indels and palindromic SNPs and retention of SNPs that overlap with those available for the target sample) we had 4,595,692 SNPs, of which 145,015 remained after clumping for use in the PRS. No *p-*value threshold from the GWAS was used for the association of SNPs to cannabis use (i.e., *p* = 1.0 was used as the threshold in PRSlice).

### Analyses

To determine the relationship of variables to each other outside of BCN modeling, we also conducted bivariate Pearson’s correlations between all variables used in BCN analyses.

Then BCN analyses were conducted using the package bnlearn [16] in R following a template of the analyses conducted in Robert et al. [18]. Code for this study is available at https://github.com/owensmax/Cannabis_Bayesian_Networks. First, a list of “blacklisted” pathways was set (Supplementary Table 2), which are pathways that cannot exist for logical reasons (e.g., age 19 cannabis use affecting sex at birth). Then the structure learning algorithms were applied to derive directed acyclic graphs representing the directional relationships between variables. To derive statistics on the BCN models, the algorithms were run 10,000 times with bootstrap re-sampling (i.e., samples drawn randomly with replacement) for samples 100% of the size of the entire dataset. From these bootstrapped BCN models a summary model is derived aggregating their results. The strength of a connection between two variables was determined as the percentage of the bootstrapped BCN models in which a connection was present. The direction of a connection was determined as the percentage of models with a connection in which the connection was directed from one variable to another. To ensure a sparse model containing only robust connections, we thresholded all models resulting from structure learning algorithms such remove connections with strength under 90%. Of note, this was primarily a choice made for visualization purposes and did not affect the structure learning process.

We conducted BCN analyses using three different types of structure learning algorithms. In score-based algorithms, the presence and direction of the relationships between variables are determined by testing numerous, randomly generated potential BCN models to evaluate which configuration of directed edges has the best model fit [31, 32]. In constraint-based algorithms, the specific tools used to determine the directional relationship between variables are statistical independence tests, which are combined with known rules about how the joint probability of three variables provides information about their directional relationship [32, 33]. In hybrid algorithms, key elements of score-based and constraint-based algorithms are combined to leverage the strengths of each approach.

To assess the consistency of results, we tested numerous structure learning algorithms from the three domains of algorithm: score-based, constraint-based, and hybrid [32, 34]. Our strategy was to extensively test algorithms from each of the three major BCN structure learning algorithm classes using a variety of options and parameters [32]. The algorithms and parameters tested are shown in Table 2. In doing this, we picked the most established algorithm in each class and created BCNs with that algorithm using a variety of approaches. Then we tested a second algorithm within these same classes using that approach’s default settings only. For the score-based models we used the hill climbing algorithm as the primary approach and the tabu algorithm as the secondary approach. For the constraint-based models we used the grow-shrink algorithm as the primary approach and the incremental association Markov blanket algorithm as the secondary approach. For the hybrid models, we used the max-min hill climbing algorithm as the primary approach and the rsmax2 algorithm as the secondary approach.

As noted, we repeated the primary analyses from the score-based algorithm approach (i.e., hill climbing) and the constraint-based algorithm approach (i.e., grow-shrink) using different values for key modifiable parameters. For the hill climbing algorithm, the specific fit index by which the model fit is assessed is critical to the validity of this approach. Our primary analysis used the Bayesian Information Criterion as the fit index, which is among the most common and interpretable of these criteria [35]. Additionally, a secondary analysis used the Akaike Information Criterion as the fit index. Another flexible parameter for the hill climbing algorithm is the starting point for model building. In our primary analysis the hill climbing algorithm started from an empty BCN, but in a separate secondary analysis the hill climbing algorithm started from a randomly generated BCN. Additionally, perturbations can be randomly introduced to the hill climbing algorithm to reduce the chance of landing at a local minimum. Thus, in another secondary analysis, we introduced random perturbations of the BCN to our primary analysis’s hill climbing procedure. For the grow-shrink algorithm, the most flexible parameter is the test used to determine dependence/independence. Our primary dependence test was the mutual information test, which determines the amount of information that can be obtained about one random variable by observing the other random variable [36]. However, in two separate secondary analyses we used two other common independence tests: the Fisher Z test and the Pearson’s correlation.

In addition to these analyses, we also built models using our primary algorithm (hill climbing) with one direction between cannabis use and DPFC thickness “blacklisted”. By testing both backlisted directions in separate models, we were able to compare the two model’s fit statistics as another means of evaluating the directional relationship between these two variables.

## Results

### Preliminary analyses

Pearson’s correlation analyses are reported in Table 1. As expected from Albaugh et al., change in cortical thickness in the dorsal prefrontal (DPFC) region of interest between ages 14 and 19 was associated with cannabis use during that same period (*r* = −0.17, *p* = 1e-5). Of note, a cannabis use PRS [29] was associated with cannabis use levels reported at age 19 (*r* = 0.10, *p* = 0.02).

**Table 1:** Pearson correlations among variables used in the Bayesian Network modeling.

	1	2	3	4	5	6	7	8	9	10	11	12	13	14	15	16
1. Baseline DPFC Thickness																
2. Change DPFC Thickness	−**0.50**															
3. Change Cannabis Use	−0.04	−**0.17**														
4. Tobacco Use Baseline	−**0.14**	0.04	**0.26**													
5. Change Tobacco Use	−0.03	−**0.09**	**0.49**	−0.05												
6. Alcohol Use Baseline	−0.06	−0.02	**0.26**	**0.39**	**0.19**											
7. Change Alcohol Use	0.03	−0.08	**0.19**	−**0.13**	**0.28**	−**0.32**										
8. Handedness	−0.02	0.03	0.01	0.05	−0.06	0	−0.02									
9. Baseline Age	−**0.13**	**0.10**	−0.03	0.04	−**0.09**	0.05	−0.05	−0.04								
10. Cannabis PRS	−0.01	−0.01	**0.10**	−0.05	**0.11**	0.06	0.07	−0.07	−0.02							
11. SES	0.06	−0.04	0.02	−**0.17**	−0.06	−0.04	**0.08**	−0.01	−0.07	0.05						
12. Pubertal Development	0	**0.09**	0	0.03	0.05	**0.17**	−0.08	−0.02	**0.23**	−0.05	0.02					
13. ADHD Baseline	0.04	−**0.11**	**0.11**	0.06	**0.13**	0.12	0.06	−0.01	−**0.08**	0.04	−**0.10**	−0.08				
14. Change ADHD	−0.01	0.04	0	−0.04	0.03	−**0.10**	0.08	−0.01	0.01	0.04	0.02	0.02	−**0.56**			
15. Childhood Trauma	−0.03	0.01	0.02	0.05	−0.01	0.01	−0.02	0.07	0.01	0.01	−**0.08**	−0.05	0	0.05		
16. SS	−**0.11**	0	**0.16**	**0.14**	**0.13**	**0.23**	0.02	−0.02	−0.04	0.01	0	**0.10**	**0.08**	−0.02	−0.03	
17. Change SS	**0.09**	−0.05	0.09	−**0.08**	0.07	−**0.10**	**0.17**	−0.03	−**0.08**	0.05	0	−0.06	0.06	0.04	0.04	−**0.49**

All variables were residualized for site and sex. Bold text indicates *p* < 0.05.

*PRS* Cannabis use polygenic risk score, *DPFC* dorsal prefrontal thickness, *SES* Socioeconomic Status, *ADHD* attention/deficit-hyperactivity disorder, *SS* sensation seeking, *Change* change in a variable from ages 14 to 19.

### Bayesian network results

**Fig. 1:**
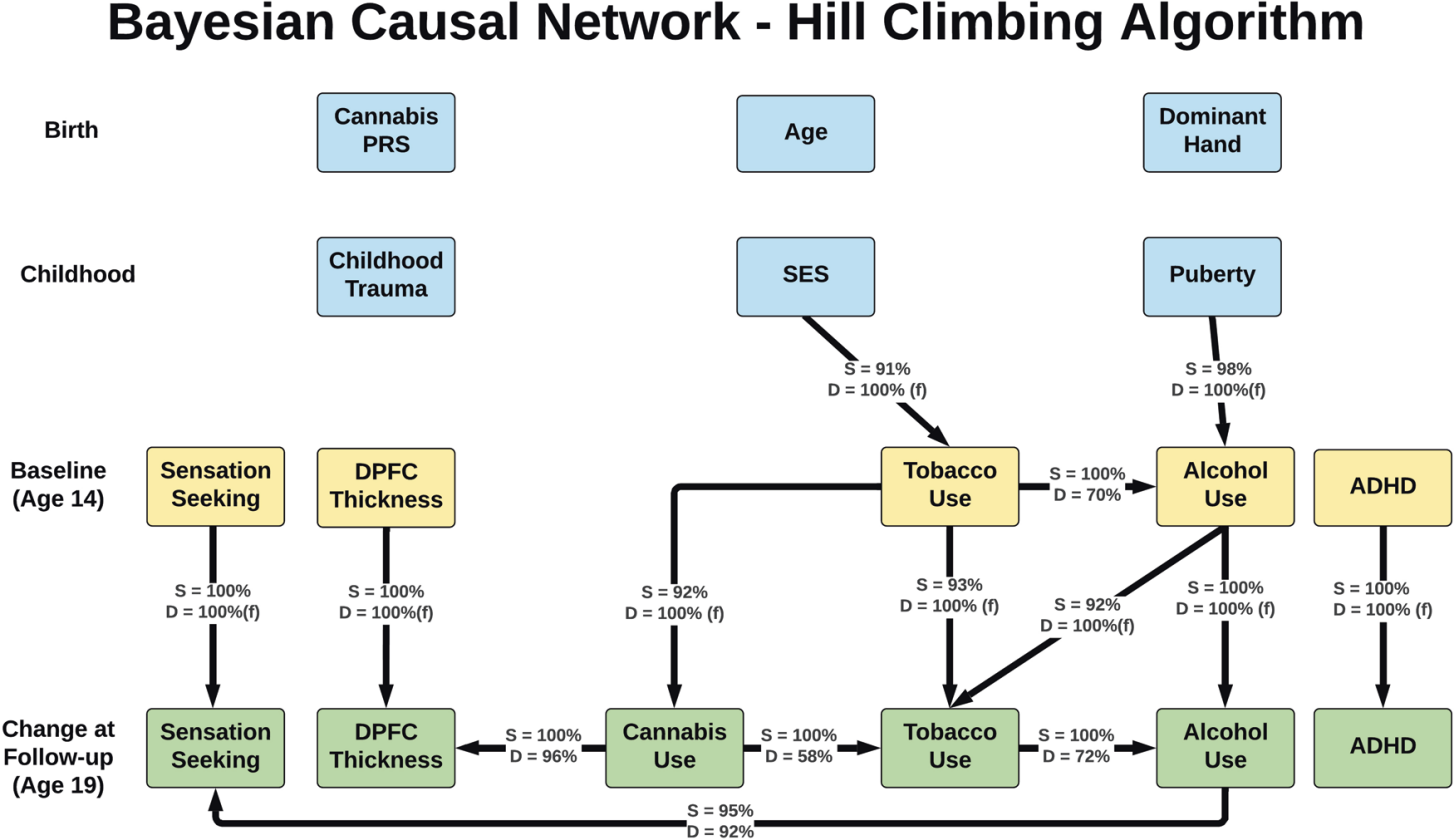
Primary Analysis: Bayesian network model from the hill climbing algorithm. Boxes represent variables used in Bayesian Causal Network models. Yellow boxes are age 14 variables, green boxes are change from age 14 to 19 variables, and blue boxes are other variables of interest. Lines indicate a dependent relationship between two variables in at least 90% of 10,000 bootstrapped models (i.e., strength ≥90%). Arrows indicate directional of relationship found between two variables. S = strength, representing the percentage of bootstrapped models in which a dependent relationship was present. D = direction, representing the percentage of bootstrapped models with a dependent relationship in which a connection was in the direction shown in the figure. (f) = connection with direction pre-specified to fit with temporal ordering. Note: all participants were cannabis-naïve at age 14. All variables were residualized for site and sex.

All BCN modeling algorithms yielded similar patterns of connections with similar coefficients. Figure 1 shows the summary model from our primary analysis, which aggregates across the 10,000 bootstrapped resampling to yield strength and direction coefficients. This model used the hill climbing algorithm starting from an empty graph with Bayesian information criterion as the goodness of fit metric and found that cannabis use was affecting DPFC thickness in 96% of models. Notably, cannabis use was the only variable directly affecting DPFC thickness. Also noteworthy, despite their relationship in bivariate correlations, the cannabis use PRS did not show a direct link to cannabis use in BCN modeling.

Strength and direction coefficients for the cannabis use to DPFC thickness connection across all models are shown in Table 2. Additionally, for all modeling approaches used, model coefficients with greater than 60% strength are reported in Supplementary Tables 3–12. Our primary model, Hill Climbing, is also visualized including all coefficients with strength greater than 60% in Supplementary Fig. 2. For all score-based algorithms, the connection from cannabis use to DPFC thickness had a strength ranging from 99% to 100% and a direction ranging from 91% to 96%. Similar results were found across constraint-based algorithms, with the connection from cannabis use to DPFC thickness having strength ranging from 91% to 97% and direction ranging from 81% to 82%. The two-hybrid algorithms, Max–min hill climbing and RSMAX2, showed comparable results to the score-based algorithms, finding strength values of 92%/93% and direction values of 95% in these algorithms. In analyses with one direction restricted (i.e., “blacklisted”) between DPFC thickness and cannabis use, the model in which cannabis use was only allowed to affect DPFC thickness had a better fit than the model in which DPFC thickness was only allowed to affect cannabis use (ΔBIC = 5.2).

**Table 2:** Summary of Bayesian causal network analytic approaches conducted.

SCORE-BASED ALGORITHMS						
Algorithm	Fit criterion	Start Point	Random Perturbations	Independence Test	Strength	Direction
*Hill Climbing	Bayesian Information Criterion	Empty Graph	No	-	100%	96%
Hill Climbing	Akaike Information Criterion	Empty Graph	No	-	100%	95%
Hill Climbing	Bayesian Information Criterion	Random Graph	No	-	99%	91%
Hill Climbing	Bayesian Information Criterion	Empty Graph	Yes	-	100%	95%
Tabu	Bayesian Information Criterion	Empty Graph	No	-	100%	95%
**CONSTRAINT-BASED ALGORITHMS**						
**Algorithm**	**Fit criterion**	**Start Point**	**Random Perturbations**	**Independence Test**	**Strength**	**Direction**
Grow-Shrink	-	-	-	Mutual Information Test	97%	82%
Grow-Shrink	-	-	-	Fisher Z Test	91%	81%
Grow-Shrink	-	-	-	Pearson Correlation	97%	82%
IAMB	-	-	-	Mutual Information Test	96%	82%
**HYBRID ALGORITHMS**						
**Algorithm**	**Fit criterion**	**Start Point**	**Random Perturbations**	**Independence Test**	**Strength**	**Direction**
Max–Min Hill Climbing	Bayesian Information Criterion	Empty Graph	No	Mutual Information Test	93%	95%
RSMAX2	Bayesian Information Criterion	Empty Graph	No	Mutual Information Test	92%	95%

“-” represents cells that are not applicable. The asterisked row (*) corresponds with the graph in Fig. 1.

## Discussion

In using BCN modeling to attempt to disentangle the directionality of the relationship between adolescent cannabis use and DPFC thinning, the current study’s results were overwhelmingly supportive of the conclusion that adolescent cannabis use affects DPFC thickness rather than the alternative hypotheses that DPFC thickness development affects an adolescent’s likelihood of beginning to use cannabis or that the two are not affecting one another. To ensure stability of the results, we tested two algorithms from each of the three BCN structure learning classes using a variety of analytic approaches for each algorithm. In addition to these bottom-up approaches to model building, we also used a top-down model building strategy that pre-specified the relationship between cannabis use and change in DPFC thickness to test which showed a better model fit. BCNs built using every algorithm and parameter combination indicated cannabis was affecting DPFC thickness, with most models finding this in over 90% of bootstrap samples and all models finding this in over 80% of bootstrap samples. Score-based and constraint-based approaches to BCN construction are sufficiently unique that their convergence in the current study adds to the confidence of these results. Thus, the consistency of results among these approaches is compelling evidence in favor of a causal relationship of adolescent cannabis use on DPFC thickness.

One notable feature of the models constructed is that the strength of the relationship between cannabis use and DPFC thickness was expected given how the region was chosen: the region of DPFC that we used was selected specifically because it was found in our previous work to be related to cannabis use [1]. Therefore, it was unsurprising that the strength of the connection between cannabis use and DPFC thinning from ages 14 to 19 was 100%. However, what is notable in the current results is that the direction of this relationship was so consistently found to go from cannabis use to cortical thickness. This was not explored in our previous analyses and represents the unique contribution of this study.

There are several relationships noteworthy for being present or absent in the current findings. Many connections identified by the BCN algorithms were expected based on existing literature. For instance, low socioeconomic status showed a directional effect on smoking at age 14, which is consistent with a robust literature linking smoking to low socioeconomic status [37]. Likewise, early pubertal development showed a directional relationship with age 14 alcohol use, as has been shown previously [38]. However, there were also expected relationships not present. One of these was a relationship between the cannabis PRS and cannabis use. Despite its bivariate association with cannabis use (*r* = 0.10), the PRS for cannabis use was not associated with cannabis use in any BCN model. This is because BCN models report the relationship between variables in the context of all other variables in the model, suggesting that other factors in the model better explain the likelihood of cannabis use. The lack of associations of the cannabis PRS with cannabis use or DPFC thickness is evidence against an alternate explanation that a pre-existing genetic trajectory causally affects DPFC thinning and cannabis use in a confounding manner. There were also some expected relationships that were not observed in our main model but were found when the strength threshold was lowered to 60%, as reported in Supplementary Tables 3–12. For example, there was a directional relationship from ADHD symptoms at age 14 to alcohol use at age 14, suggesting that ADHD may have a weak directional effect on alcohol use. Further, it should be noted that the current results differ from the observed relationship between alcohol use and brain structure using BCN structure learning algorithms conducted by Robert et al. [18]. In that study, BCN structure learning algorithms overwhelmingly indicated that volume reduction in the prefrontal and temporal cortices was causally affected by alcohol consumption in adolescents from the IMAGEN study. The discrepancy between that study and the current one illustrates that BCN algorithms can arrive at quite different results in similar situations.

Given their importance to the current study, it is worth considering how BCN structure learning algorithms estimate the directional relationships between variables. These algorithms apply an emerging understanding of how directional relationships are predicated on conditional dependence to determine the BCN that best represents the joint probability distributions of a dataset [16]. In score-based algorithms, the presence and direction of the relationships between variables are determined by testing numerous, randomly generated potential BCN models to evaluate which configuration of directed edges has the best model fit [31, 32]. In constraint-based algorithms, the specific tools used to determine the directional relationship between variables are statistical independence tests, which are combined with known rules about how the joint probability of three variables provides information about their directional relationship [32, 33]. In hybrid algorithms, key elements of score-based and constraint-based algorithms are combined to leverage the strengths of each approach. Score-based and constraint-based approaches to BCN construction are sufficiently unique that their convergence in the current study adds to our confidence in these results. Within the assumptions and constraints of the methodology, BCN models offer a complementary approach to infer probable causal associations from amidst the complex, confounded, and colinear measurements that so typically characterizes nonexperimental human research.

One important limitation of these findings is that BCN modeling estimates the direction of a relationship only in the context of the variables included in its models: BCN modeling is not able to detect hidden or unmeasured variables that may be affecting the variables within the model and, consequently, like most statistical methods, is susceptible to unmeasured confounders. The present analysis did include many relevant variables in the BCN model that represent obvious potential confounding factors, including demographics, genetics, psychopathology, personality, childhood adversity, and measures of other substance use. While there are, of course, an infinitely large number of potential confounders, the current analysis suggests that in the context of many of the most likely confounders, there is a directional relationship from adolescent cannabis use to dorsal prefrontal cortical thickness that is consistent with causality. However, it remains possible that some unmeasured confounder could alter these results. Considering this, we suggest that the best way to conceive of the present approach is that it addresses a central, but unresolved, question on the directionality of the previously observed association between cannabis use and dorsal prefrontal cortex thickness, but that it does so within the confines of the specific (and necessarily finite) number of confounders that were included. While certainly not definitive, the present results provide a thorough initial test of this question using BCNs, finding results highly supportive of a causal effect of cannabis on brain development. We hope that future work, whether employing causal modeling or not, further investigates this matter and continues to evaluate other potential confounding variables.

Another consideration is that the association between DPFC thinning and cannabis use was still relatively small, *r* = 0.17 in bivariate analyses. This suggests that there is much more than cannabis use that goes into cortical development and other important psychological and social contributors to development should not be overlooked. Of note, cannabis use was assessed in the current study using a retrospective, self-report measure and its possible that with a more thorough measure (e.g., ecological momentary assessment of cannabis use) the effect sizes seen in bivariate analyses may have been larger. However, a correlation of 0.17 is not insignificant either, particularly when compared to other meaningful effect sizes found in similar datasets [39].

Prior research has estimated normative cortical thinning of around 1% annually, which comes out to around 0.03–0.06 mm per year [8–11]. Results of the current study were highly consistent with this prior literature, as the average percent reduction in cortical thickness from ages 14 to 19 was 3.6% (i.e., 0.12 mm reduction in thickness). In cannabis-using participants, the average percent reduction was 4.4% (0.14 mm reduction in thickness), compared to an average reduction of 3.1% in cannabis abstinent participants (a 0.10 mm reduction in thickness). The heaviest using participants (those who used cannabis 40 or more times between the ages of 14 and 19) demonstrated a 5% reduction in cortical thickness (i.e., 0.17 mm reduction in thickness), while the lightest using participants (those reporting fewer than 10 uses between the ages of 14 and 19) showed a 4.1% reduction (0.13 mm). Compared to those who did not use cannabis, this represents a 30% greater reduction in cortical thickness in all cannabis-using participants, a 24% greater reduction in the lightest using participants, and a 38% greater reduction in the heaviest using participants. Notably, differences in rate of thinning comparable to those found in the current manuscript have been noted as signifying greater risk of depression in adolescents in other work [14]. Thus, while noting that the quantification of cortical thinning is imperfect, resting on MRI contrast images [40], we think that these findings do suggest an association sufficiently large to merit concern.

The present results report a notable association between DPFC thinning and cannabis use in a dose-dependent manner. While noting again the caveats associated with inferring causation from human research, these findings complement other humans [1, 3, 4, 7, 41] and animal [5] research and add to the increasingly compelling evidence base that adolescent cannabis use affects brain development.

## Supplementary information

Supplemental Materials
