# Supplementary material for: Bayesian causal network modeling suggests adolescent cannabis use accelerates prefrontal cortical thinning
Source: Transl Psychiatry. 2022 May 6;12:188. doi: 10.1038/s41398-022-01956-4 (PMC9076659; doi:10.1038/s41398-022-01956-4)

**SUPPLEMENTARY TABLES**

**Supplementary Table 1.** Strength and direction of Bayesian causal network link from “change in cannabis use from age 14 to 19” to “change in DPFC thickness” across algorithms in analyses using only the covariates from Albaugh et al., which allowed for testing models in the full sample from that study (*N* = 799).

| Algorithm | Version | Strength | Direction |
| --- | --- | --- | --- |
| Hill Climbing | Primary (fit criterion = BIC) | 96% | 92% |
| Hill Climbing | Fit criterion = AIC | 99% | 87% |
| Hill Climbing | Random start points | 98% | 94% |
| Hill Climbing | Random perturbations | 96% | 89% |
| Tabu | Default | 96% | 91% |
| Grow Shrink | Primary (Ind test = Mutual Information) | 86% | 71% |
| Grow Shrink | Ind test = Fisher Z | 78% | 74% |
| Grow Shrink | Ind test = Pearson correlation | 86% | 71% |
| IAMB | Default | 85% | 71% |
| MaxMinHC | Primary | 72% | 91% |
| RSMAX2 | Default | 72% | 92% |

**Supplemental Table 2.** List of blacklisted pathways in all Bayesian Causal Network models.

| **From** | **To** |
| --- | --- |
| Change in Alcohol Use | Cannabis Use PRS |
| Change in dPFC Thickness | Cannabis Use PRS |
| Cannabis Use by Age 19 | Cannabis Use PRS |
| Change in Tobacco Use | Cannabis Use PRS |
| Baseline dPFC Thickness | Cannabis Use PRS |
| Baseline Alcohol Use | Cannabis Use PRS |
| Baseline Tobacco Use | Cannabis Use PRS |
| Handedness | Cannabis Use PRS |
| Baseline Age | Cannabis Use PRS |
| SES | Cannabis Use PRS |
| Baseline Puberty | Cannabis Use PRS |
| CTQ total | Cannabis Use PRS |
| Baseline ADHD | Cannabis Use PRS |
| Change ADHD | Cannabis Use PRS |
| Baseline Sensation Seeking | Cannabis Use PRS |
| Change Sensation Seeking | Cannabis Use PRS |
| Change in Alcohol Use | Handedness |
| Change in dPFC Thickness | Handedness |
| Cannabis Use by Age 19 | Handedness |
| Change in Tobacco Use | Handedness |
| Baseline dPFC Thickness | Handedness |
| SES | Handedness |
| Baseline Puberty | Handedness |
| CTQ total | Handedness |
| Baseline ADHD | Handedness |
| Change ADHD | Handedness |
| Cannabis Use PRS | Handedness |
| Baseline Sensation Seeking | Handedness |
| Change Sensation Seeking | Handedness |
| Change in Alcohol Use | Baseline Age |
| Change in dPFC Thickness | Baseline Age |
| Cannabis Use by Age 19 | Baseline Age |
| Change in Tobacco Use | Baseline Age |
| Baseline dPFC Thickness | Baseline Age |
| SES | Baseline Age |
| Baseline Puberty | Baseline Age |
| CTQ total | Baseline Age |
| Baseline ADHD | Baseline Age |
| Change ADHD | Baseline Age |
| Cannabis Use PRS | Baseline Age |
| Baseline Sensation Seeking | Baseline Age |
| Change Sensation Seeking | Baseline Age |
| Change in Alcohol Use | SES |
| Change in dPFC Thickness | SES |
| Cannabis Use by Age 19 | SES |
| Change in Tobacco Use | SES |
| Baseline dPFC Thickness | SES |
| Baseline Alcohol Use | SES |
| Baseline Tobacco Use | SES |
| Handedness | SES |
| Baseline Age | SES |
| Baseline Puberty | SES |
| CTQ total | SES |
| Baseline ADHD | SES |
| Change ADHD | SES |
| Baseline Sensation Seeking | SES |
| Change Sensation Seeking | SES |
| Change in Alcohol Use | CTQ total |
| Change in dPFC Thickness | CTQ total |
| Cannabis Use by Age 19 | CTQ total |
| Change in Tobacco Use | CTQ total |
| Baseline dPFC Thickness | CTQ total |
| Baseline Alcohol Use | CTQ total |
| Baseline Tobacco Use | CTQ total |
| Handedness | CTQ total |
| Baseline Age | CTQ total |
| Baseline ADHD | CTQ total |
| Change ADHD | CTQ total |
| Baseline Puberty | CTQ total |
| Baseline Sensation Seeking | CTQ total |
| Change Sensation Seeking | CTQ total |
| Change in Alcohol Use | Baseline Puberty |
| Change in dPFC Thickness | Baseline Puberty |
| Cannabis Use by Age 19 | Baseline Puberty |
| Change in Tobacco Use | Baseline Puberty |
| Baseline dPFC Thickness | Baseline Puberty |
| Baseline Alcohol Use | Baseline Puberty |
| Baseline Tobacco Use | Baseline Puberty |
| Handedness | Baseline Puberty |
| Baseline Age | Baseline Puberty |
| CTQ total | Baseline Puberty |
| Baseline ADHD | Baseline Puberty |
| Change ADHD | Baseline Puberty |
| Baseline Sensation Seeking | Baseline Puberty |
| Change Sensation Seeking | Baseline Puberty |
| Change in Alcohol Use | Baseline ADHD |
| Change in dPFC Thickness | Baseline ADHD |
| Cannabis Use by Age 19 | Baseline ADHD |
| Change in Tobacco Use | Baseline ADHD |
| Change ADHD | Baseline ADHD |
| Change Sensation Seeking | Baseline ADHD |
| Change in Alcohol Use | Baseline dPFC Thickness |
| Change in dPFC Thickness | Baseline dPFC Thickness |
| Cannabis Use by Age 19 | Baseline dPFC Thickness |
| Change in Tobacco Use | Baseline dPFC Thickness |
| Change ADHD | Baseline dPFC Thickness |
| Change Sensation Seeking | Baseline dPFC Thickness |
| Change in Alcohol Use | Baseline Alcohol Use |
| Change in dPFC Thickness | Baseline Alcohol Use |
| Cannabis Use by Age 19 | Baseline Alcohol Use |
| Change in Tobacco Use | Baseline Alcohol Use |
| Change ADHD | Baseline Alcohol Use |
| Change Sensation Seeking | Baseline Alcohol Use |
| Change in Alcohol Use | Baseline Tobacco Use |
| Change in dPFC Thickness | Baseline Tobacco Use |
| Cannabis Use by Age 19 | Baseline Tobacco Use |
| Change in Tobacco Use | Baseline Tobacco Use |
| Change ADHD | Baseline Tobacco Use |
| Change Sensation Seeking | Baseline Tobacco Use |
| Change in Alcohol Use | Baseline Sensation Seeking |
| Change in dPFC Thickness | Baseline Sensation Seeking |
| Cannabis Use by Age 19 | Baseline Sensation Seeking |
| Change in Tobacco Use | Baseline Sensation Seeking |
| Change ADHD | Baseline Sensation Seeking |
| Change Sensation Seeking | Baseline Sensation Seeking |

**Supplemental Table 3.** Bayesian causal network model connections for Hill Climbing algorithm from primary analysis. Thresholded at 60%.

| **From** | **To** | **Strength** | **Direction** |
| --- | --- | --- | --- |
| Baseline dPFC Thickness | Change in dPFC Thickness | 100% | 100% |
| Cannabis Use by Age 19 | Change in dPFC Thickness | 100% | 96% |
| Cannabis Use by Age 19 | Change in Tobacco Use | 100% | 58% |
| Cannabis Use by Age 19 | Change in Alcohol Use | 86% | 86% |
| Cannabis Use by Age 19 | Change Sensation Seeking | 87% | 97% |
| Baseline Tobacco Use | Baseline dPFC Thickness | 82% | 72% |
| Baseline Tobacco Use | Cannabis Use by Age 19 | 92% | 100% |
| Baseline Tobacco Use | Change in Tobacco Use | 93% | 100% |
| Baseline Tobacco Use | Baseline Alcohol Use | 100% | 73% |
| Change in Tobacco Use | Change in Alcohol Use | 100% | 72% |
| Baseline Alcohol Use | Change in Tobacco Use | 92% | 100% |
| Baseline Alcohol Use | Change in Alcohol Use | 100% | 100% |
| Baseline Alcohol Use | Baseline Sensation Seeking | 98% | 97% |
| Change in Alcohol Use | Change Sensation Seeking | 95% | 92% |
| Baseline Age | Baseline dPFC Thickness | 75% | 100% |
| SES | Baseline Tobacco Use | 91% | 100% |
| Baseline Puberty | Baseline Alcohol Use | 98% | 100% |
| Baseline ADHD | Baseline Alcohol Use | 71% | 51% |
| Baseline ADHD | Change ADHD | 100% | 100% |
| Baseline Sensation Seeking | Change Sensation Seeking | 100% | 100% |

**Supplemental Table 4.** Bayesian causal network model connections for Hill Climbing algorithm using Akaike Information Criterion as fit score. Thresholded at 60%.

| **From** | **To** | **Strength** | **Direction** |
| --- | --- | --- | --- |
| Baseline dPFC Thickness | Change in dPFC Thickness | 100% | 100% |
| Baseline dPFC Thickness | Baseline Sensation Seeking | 89% | 65% |
| Cannabis Use by Age 19 | Change in dPFC Thickness | 100% | 95% |
| Cannabis Use by Age 19 | Change in Tobacco Use | 100% | 71% |
| Cannabis Use by Age 19 | Change in Alcohol Use | 95% | 84% |
| Cannabis Use by Age 19 | Change Sensation Seeking | 97% | 95% |
| Baseline Tobacco Use | Baseline dPFC Thickness | 98% | 73% |
| Baseline Tobacco Use | Cannabis Use by Age 19 | 99% | 100% |
| Baseline Tobacco Use | Change in Tobacco Use | 100% | 100% |
| Baseline Tobacco Use | Baseline Alcohol Use | 100% | 82% |
| Change in Tobacco Use | Change in Alcohol Use | 100% | 67% |
| Change in Tobacco Use | Change ADHD | 71% | 92% |
| Baseline Alcohol Use | Cannabis Use by Age 19 | 80% | 100% |
| Baseline Alcohol Use | Change in Tobacco Use | 98% | 100% |
| Baseline Alcohol Use | Change in Alcohol Use | 100% | 100% |
| Baseline Alcohol Use | Baseline ADHD | 92% | 63% |
| Baseline Alcohol Use | Baseline Sensation Seeking | 100% | 89% |
| Change in Alcohol Use | Change ADHD | 73% | 94% |
| Change in Alcohol Use | Change Sensation Seeking | 99% | 85% |
| Baseline Age | Baseline dPFC Thickness | 95% | 100% |
| Baseline Age | Change in Tobacco Use | 85% | 100% |
| Baseline Age | Baseline Sensation Seeking | 65% | 100% |
| Baseline Age | Change Sensation Seeking | 87% | 100% |
| Cannabis Use PRS | Cannabis Use by Age 19 | 71% | 100% |
| Cannabis Use PRS | Baseline Alcohol Use | 82% | 100% |
| SES | Cannabis Use by Age 19 | 66% | 100% |
| SES | Baseline Tobacco Use | 99% | 100% |
| SES | Change in Tobacco Use | 88% | 100% |
| SES | Change in Alcohol Use | 72% | 100% |
| SES | Baseline ADHD | 88% | 100% |
| SES | CTQ total | 73% | 100% |
| Baseline Puberty | Change in dPFC Thickness | 85% | 100% |
| Baseline Puberty | Baseline Alcohol Use | 100% | 100% |
| Baseline Puberty | Baseline ADHD | 63% | 100% |
| Baseline Puberty | Baseline Sensation Seeking | 68% | 100% |
| Baseline ADHD | Change in Alcohol Use | 60% | 100% |
| Baseline ADHD | Change ADHD | 100% | 100% |
| Baseline ADHD | Change Sensation Seeking | 72% | 100% |
| Baseline Sensation Seeking | Cannabis Use by Age 19 | 69% | 100% |
| Baseline Sensation Seeking | Change in Alcohol Use | 60% | 100% |
| Baseline Sensation Seeking | Change Sensation Seeking | 100% | 100% |
| Change Sensation Seeking | Change ADHD | 72% | 52% |

**Supplemental Table 5.** Bayesian causal network model connections for Hill Climbing algorithm using random perturbations. Thresholded at 60%.

| **From** | **To** | **Strength** | **Direction** |
| --- | --- | --- | --- |
| Baseline dPFC Thickness | Change in dPFC Thickness | 100% | 100% |
| Cannabis Use by Age 19 | Change in dPFC Thickness | 100% | 95% |
| Cannabis Use by Age 19 | Change in Tobacco Use | 100% | 57% |
| Cannabis Use by Age 19 | Change in Alcohol Use | 86% | 85% |
| Cannabis Use by Age 19 | Change Sensation Seeking | 87% | 97% |
| Baseline Tobacco Use | Baseline dPFC Thickness | 82% | 72% |
| Baseline Tobacco Use | Cannabis Use by Age 19 | 92% | 100% |
| Baseline Tobacco Use | Change in Tobacco Use | 94% | 100% |
| Baseline Tobacco Use | Baseline Alcohol Use | 100% | 73% |
| Change in Tobacco Use | Change in Alcohol Use | 100% | 72% |
| Baseline Alcohol Use | Change in Tobacco Use | 92% | 100% |
| Baseline Alcohol Use | Change in Alcohol Use | 100% | 100% |
| Baseline Alcohol Use | Baseline Sensation Seeking | 98% | 97% |
| Change in Alcohol Use | Change Sensation Seeking | 96% | 92% |
| Baseline Age | Baseline dPFC Thickness | 74% | 100% |
| SES | Baseline Tobacco Use | 91% | 100% |
| Baseline Puberty | Baseline Alcohol Use | 98% | 100% |
| Baseline ADHD | Baseline Alcohol Use | 71% | 51% |
| Baseline ADHD | Change ADHD | 100% | 100% |
| Baseline Sensation Seeking | Change Sensation Seeking | 100% | 100% |

**Supplemental Table 6.** Bayesian causal network model connections for the Tabu Algorithm. Thresholded at 60%.

| **From** | **To** | **Strength** | **Direction** |
| --- | --- | --- | --- |
| Baseline dPFC Thickness | Change in dPFC Thickness | 100% | 100% |
| Cannabis Use by Age 19 | Change in dPFC Thickness | 100% | 95% |
| Cannabis Use by Age 19 | Change in Tobacco Use | 100% | 56% |
| Cannabis Use by Age 19 | Change in Alcohol Use | 86% | 83% |
| Cannabis Use by Age 19 | Change Sensation Seeking | 87% | 96% |
| Baseline Tobacco Use | Baseline dPFC Thickness | 82% | 73% |
| Baseline Tobacco Use | Cannabis Use by Age 19 | 93% | 100% |
| Baseline Tobacco Use | Change in Tobacco Use | 94% | 100% |
| Baseline Tobacco Use | Baseline Alcohol Use | 100% | 71% |
| Change in Tobacco Use | Change in Alcohol Use | 100% | 69% |
| Baseline Alcohol Use | Change in Tobacco Use | 93% | 100% |
| Baseline Alcohol Use | Change in Alcohol Use | 100% | 100% |
| Baseline Alcohol Use | Baseline Sensation Seeking | 98% | 96% |
| Change in Alcohol Use | Change Sensation Seeking | 95% | 90% |
| Baseline Age | Baseline dPFC Thickness | 77% | 100% |
| SES | Baseline Tobacco Use | 91% | 100% |
| SES | Change in Tobacco Use | 60% | 100% |
| Baseline Puberty | Baseline Alcohol Use | 98% | 100% |
| Baseline ADHD | Baseline Alcohol Use | 72% | 52% |
| Baseline ADHD | Change ADHD | 100% | 100% |
| Baseline Sensation Seeking | Change Sensation Seeking | 100% | 100% |

**Supplemental Table 7.** Bayesian causal network model connections for primary Grow Shrink algorithm. Thresholded at 60%

| **From** | **To** | **Strength** | **Direction** |
| --- | --- | --- | --- |
| Baseline dPFC Thickness | Change in dPFC Thickness | 100% | 100% |
| Cannabis Use by Age 19 | Change in dPFC Thickness | 97% | 82% |
| Baseline Tobacco Use | Baseline dPFC Thickness | 65% | 64% |
| Baseline Tobacco Use | Cannabis Use by Age 19 | 96% | 100% |
| Baseline Tobacco Use | Baseline Alcohol Use | 100% | 54% |
| Change in Tobacco Use | Cannabis Use by Age 19 | 100% | 93% |
| Change in Tobacco Use | Change in Alcohol Use | 99% | 85% |
| Baseline Alcohol Use | Change in Alcohol Use | 100% | 100% |
| Baseline Alcohol Use | Baseline Sensation Seeking | 91% | 75% |
| Change in Alcohol Use | Change Sensation Seeking | 86% | 78% |
| Baseline Age | Baseline dPFC Thickness | 62% | 100% |
| SES | Baseline Tobacco Use | 95% | 100% |
| SES | Baseline ADHD | 60% | 100% |
| Baseline Puberty | Baseline Alcohol Use | 89% | 100% |
| ADHD Basline | Change ADHD | 100% | 100% |
| Baseline Sensation Seeking | Change Sensation Seeking | 100% | 100% |

**Supplemental Table 8.** Bayesian causal network model connections for Grow Shrink algorithm using Fisher Z Test as for independence tests. Thresholded at 60%

| **From** | **To** | **Strength** | **Direction** |
| --- | --- | --- | --- |
| Baseline dPFC Thickness | Change in dPFC Thickness | 100% | 100% |
| Cannabis Use by Age 19 | Change in dPFC Thickness | 91% | 81% |
| Baseline Tobacco Use | Cannabis Use by Age 19 | 90% | 100% |
| Change in Tobacco Use | Cannabis Use by Age 19 | 100% | 92% |
| Change in Tobacco Use | Change in Alcohol Use | 98% | 80% |
| Baseline Alcohol Use | Change in Alcohol Use | 100% | 100% |
| Baseline Alcohol Use | Baseline Sensation Seeking | 83% | 74% |
| Change in Alcohol Use | Change Sensation Seeking | 76% | 86% |
| SES | Baseline Tobacco Use | 83% | 100% |
| Baseline Puberty | Baseline Alcohol Use | 73% | 100% |
| Baseline ADHD | change ADHD | 100% | 100% |

**Supplemental Table 9.** Bayesian causal network model connections for Grow Shrink algorithm using Pearson’s correlation as for independence tests. Thresholded at 60%

| **From** | **To** | **Strength** | **Direction** |
| --- | --- | --- | --- |
| Baseline dPFC Thickness | Change in dPFC Thickness | 100% | 100% |
| Cannabis Use by Age 19 | Change in dPFC Thickness | 97% | 82% |
| Baseline Tobacco Use | Cannabis Use by Age 19 | 96% | 100% |
| Change in Tobacco Use | Cannabis Use by Age 19 | 100% | 92% |
| Change in Tobacco Use | Change in Alcohol Use | 99% | 85% |
| Baseline Alcohol Use | Change in Alcohol Use | 100% | 100% |
| Baseline Alcohol Use | Baseline Sensation Seeking | 90% | 72% |
| Change in Alcohol Use | Change Sensation Seeking | 86% | 84% |
| Baseline Age | Baseline dPFC Thickness | 62% | 100% |
| SES | Baseline Tobacco Use | 95% | 100% |
| SES | Baseline ADHD | 60% | 100% |
| Baseline Puberty | Baseline Alcohol Use | 88% | 100% |
| Baseline ADHD | Change ADHD | 100% | 100% |
| Baseline Sensation Seeking | Change Sensation Seeking | 100% | 74% |

**Supplemental Table 10.** Bayesian causal network model connections for IAMB algorithm. Thresholded at 60%.

| **From** | **To** | **Strength** | **Direction** |
| --- | --- | --- | --- |
| Baseline dPFC Thickness | Change in dPFC Thickness | 100% | 100% |
| Cannabis Use by Age 19 | Change in dPFC Thickness | 96% | 82% |
| Baseline Tobacco Use | Baseline dPFC Thickness | 64% | 64% |
| Baseline Tobacco Use | Cannabis Use by Age 19 | 96% | 100% |
| Baseline Tobacco Use | Baseline Alcohol Use | 100% | 54% |
| Change in Tobacco Use | Cannabis Use by Age 19 | 100% | 93% |
| Change in Tobacco Use | Change in Alcohol Use | 99% | 84% |
| Baseline Alcohol Use | Change in Alcohol Use | 100% | 100% |
| Baseline Alcohol Use | Baseline Sensation Seeking | 91% | 71% |
| Change in Alcohol Use | Change Sensation Seeking | 85% | 83% |
| Baseline Age | Baseline dPFC Thickness | 61% | 100% |
| SES | Baseline Tobacco Use | 94% | 100% |
| SES | Baseline ADHD | 61% | 100% |
| Baseline Puberty | Baseline Alcohol Use | 88% | 100% |
| Baseline ADHD | Change ADHD | 100% | 100% |
| Baseline Sensation Seeking | Change Sensation Seeking | 100% | 74% |

**Supplemental Table 11.** Bayesian causal network model connections for Max-Min Hill Climbing algorithm. Thresholded at 60%

| **From** | **To** | **Strength** | **Direction** |
| --- | --- | --- | --- |
| Baseline dPFC Thickness | Change in dPFC Thickness | 100% | 100% |
| Cannabis Use by Age 19 | Change in dPFC Thickness | 93% | 95% |
| Baseline Tobacco Use | Baseline dPFC Thickness | 78% | 73% |
| Baseline Tobacco Use | Cannabis Use by Age 19 | 94% | 100% |
| Baseline Tobacco Use | Baseline Alcohol Use | 100% | 66% |
| Change in Tobacco Use | Cannabis Use by Age 19 | 100% | 86% |
| Change in Tobacco Use | Change in Alcohol Use | 100% | 93% |
| Baseline Alcohol Use | Change in Alcohol Use | 100% | 100% |
| Baseline Alcohol Use | Baseline Sensation Seeking | 90% | 95% |
| Change in Alcohol Use | Change Sensation Seeking | 83% | 99% |
| SES | Baseline Tobacco Use | 90% | 100% |
| Baseline Puberty | Baseline Alcohol Use | 87% | 100% |
| Baseline ADHD | Change ADHD | 100% | 100% |
| Baseline Sensation Seeking | Change Sensation Seeking | 100% | 100% |

**Supplemental Table 12.** Bayesian causal network model connections for Max-Min Hill Climbing algorithm. Thresholded at 60%

| **From** | **To** | **Strength** | **Direction** |
| --- | --- | --- | --- |
| Baseline dPFC Thickness | Change in dPFC Thickness | 100% | 100% |
| Cannabis Use by Age 19 | Change in dPFC Thickness | 92% | 95% |
| Baseline Tobacco Use | Baseline dPFC Thickness | 79% | 73% |
| Baseline Tobacco Use | Cannabis Use by Age 19 | 94% | 100% |
| Baseline Tobacco Use | Baseline Alcohol Use | 100% | 65% |
| Change in Tobacco Use | Cannabis Use by Age 19 | 100% | 85% |
| Change in Tobacco Use | Change in Alcohol Use | 100% | 94% |
| Baseline Alcohol Use | Change in Alcohol Use | 100% | 100% |
| Baseline Alcohol Use | Baseline Sensation Seeking | 90% | 95% |
| Change in Alcohol Use | Change Sensation Seeking | 83% | 98% |
| SES | Baseline Tobacco Use | 89% | 100% |
| Baseline Puberty | Baseline Alcohol Use | 90% | 100% |
| Baseline ADHD | Change ADHD | 100% | 100% |
| Baseline Sensation Seeking | Change Sensation Seeking | 100% | 96% |

**Supplementary Figure 1.** Primary Analysis repeated using only the covariates from Albaugh et al., which allowed for testing models in the full sample from that study (*N* = 799). Bayesian network model from Hill Climbing algorithm. Boxes represent variables used in Bayesian Causal Network models. Yellow boxes are Baseline variables, green boxes are change from Baseline to follow-up variables, and blue boxes are other variables of interest. Lines indicate a dependent relationship between two variables in at least 90% of 10,000 bootstrapped models (i.e., strength ≥ 90%). Arrows indicate directional relationship found in at least 70% of models with a dependent relationship between two variables (i.e., direction ≥ 70%). Absence of a line indicates two variables are dependent in less than 90% of models. S = strength, representing the percentage of bootstrapped models in which a dependent relationship is present. D = direction, representing the percentage of bootstrapped models with a dependent relationship in which a connection is in the direction shown in the figure. (f) = connection with direction pre-specified to fit with temporal ordering. Note: all participants were cannabis-naïve at age 14. All variables were residualized for site and sex.

**
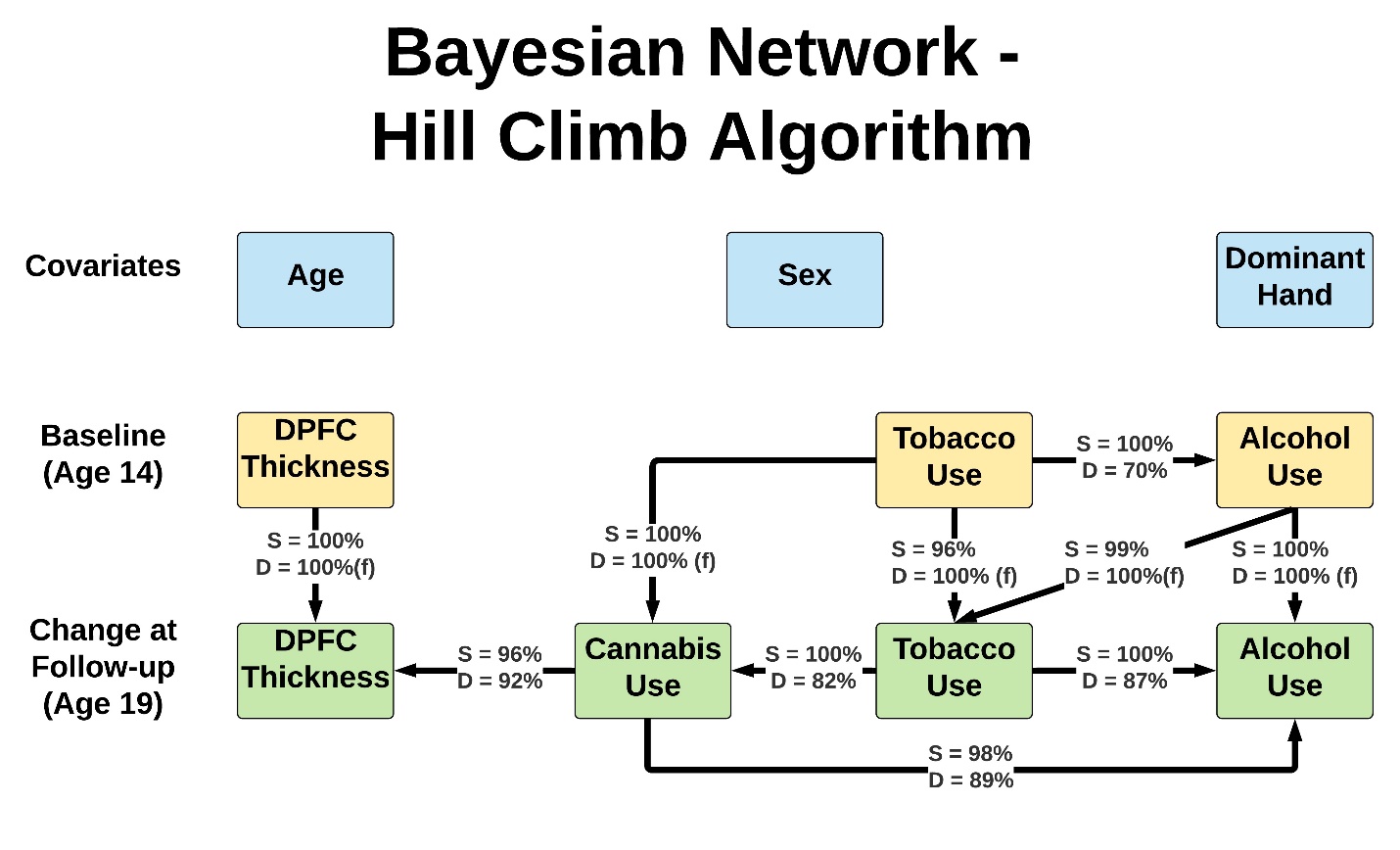
**

**Supplemental Figure 2.** Primary Analysis: Bayesian network model from the hill climbing algorithm using a lower threshold (60% strength). Boxes represent variables used in Bayesian Causal Network models. Yellow boxes are age 14 variables, green boxes are change from age 14 to 19 variables, and blue boxes are other variables of interest. Lines indicate a dependent relationship between two variables in at least 60% of 10,000 bootstrapped models (i.e., strength ≥ 60%). Arrows indicate directional of relationship found between two variables. S = strength, representing the percentage of bootstrapped models in which a dependent relationship was present. D = direction, representing the percentage of bootstrapped models with a dependent relationship in which a connection was in the direction shown in the figure. (f) = connection with direction pre-specified to fit with temporal ordering. Note: all participants were cannabis-naïve at age 14. All variables were residualized for site and sex.


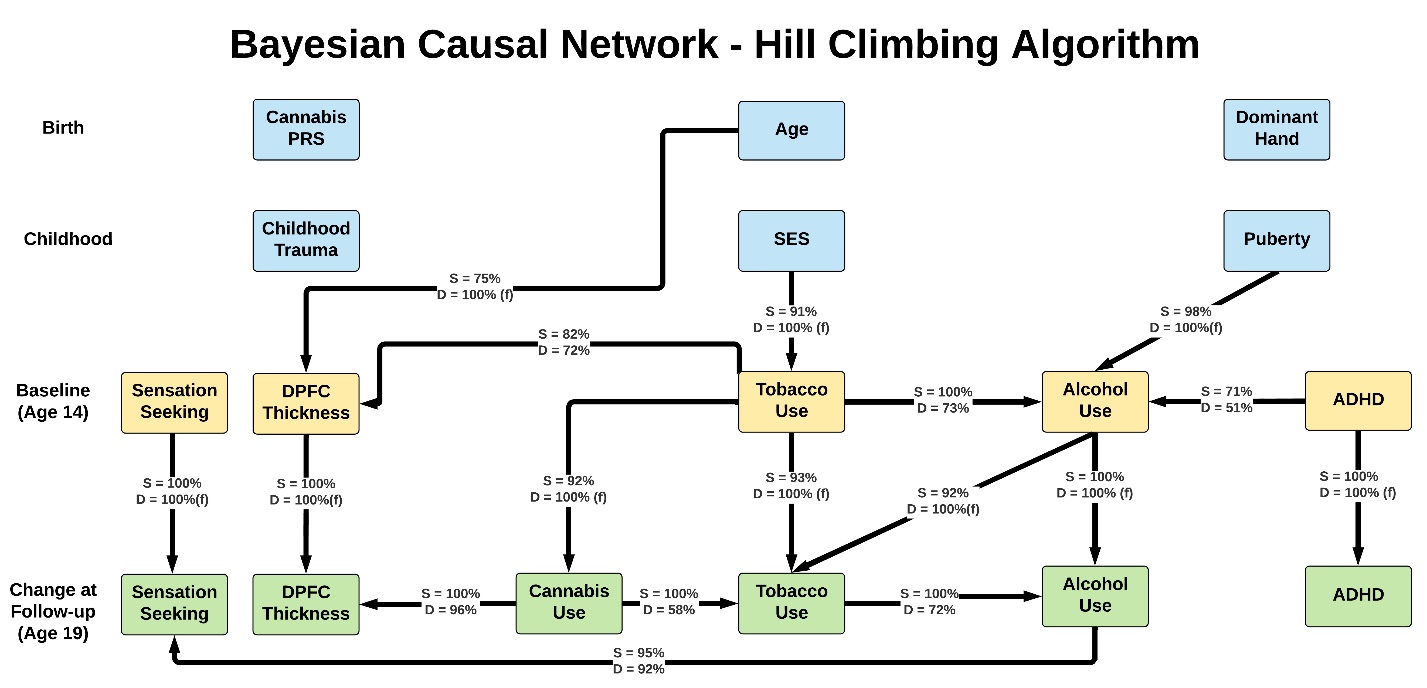

Supplement: Supplementary file 1 — Supplemental Materials [file 41398_2022_1956_MOESM1_ESM.docx]
